# Strengthening integrated sexual reproductive health and rights and HIV services programs to achieve sustainable development goals 3 and 5 in Africa

**DOI:** 10.1186/s12978-022-01535-2

**Published:** 2022-12-09

**Authors:** Rose-Mary Asong Tazinya, Julia Marie Hajjar, Sanni Yaya

**Affiliations:** 1 Interdisciplinary School of Health Sciences, University of Ottawa, Ottawa, Canada; 2 School of International Development and Global Studies, Faculty of Social Sciences, University of Ottawa, 120 University Private, Ottawa, ON K1N 6N5 Canada; 3 The George Institute for Global Health, Imperial College London, London, UK

## Abstract

Each year, over 200 million women globally cannot prevent pregnancy through modern contraceptive methods, with 70–80% of these women residing in sub-Saharan Africa. Consequently, almost 50% of pregnancies are unintended and 35 million unsafe abortions occur annually in the region. Further, sub-Saharan Africa has the highest burden globally of Human Immune-Deficiency Virus (HIV) infection, and over 57% of those affected are women. Women with a positive HIV status in sub-Saharan Africa experience higher rates of unintended pregnancy and unsafe abortion practices. In this commentary, we propose strategies to strengthen integrated sexual and reproductive health and rights (SRHR) and HIV services programs to improve the sexual and reproductive health of girls and women and to work towards achieving SDGs 3 and 5 in sub-Saharan Africa. We suggest a focus on capacity building, strengthening intersectoral collaborations, and improving governance and financial investment.

## Introduction

Every year globally, more than 200 million women are not able to meet their needs to prevent pregnancy through modern contraception [1], with about 70–80% living in sub-Saharan Africa [2]. This leads to a high rate of unintended pregnancies in this region (49%) and about 35 million unsafe abortions annually [3]. Up to 45 million women receive none or suboptimal antenatal care coverage worldwide [1] with the majority living in low and middle-income countries (LMICs). This consequently increases the risk of women dying from pregnancy-related complications in these developing countries to 1 in 26, an astounding number, compared to 1 in 7300 in the developed world [2]. Notably, Sub-Saharan Africa has the highest burden of Human Immunodeficiency Virus (HIV) infections in the world and women constitute about 57% of the persons affected [4]. Early sexual activity is an important factor that can increase the likelihood of becoming HIV positive [6, 7]. Notably, HIV-positive women tend to practice lower condom use compared to their peers who are HIV negative [5]. Consequently, women who are positive for HIV experience higher rates of unwanted pregnancies and unsafe abortion practices [5]. This highlights an evident need for additional programs and services tailored to the sexual and reproductive health needs of HIV-positive women. Offering integrated services that address sexual and reproductive health and rights (SRHR) with HIV-related programs and services will more effectively support women to make more informed and empowered decisions regarding their reproductive health choices. In this commentary, we provide several recommendations on what is needed to effectively integrate these services.

### Sustainable development goals (SDGs) in LMICs

The sustainable development goals (SDGs) were drafted after the United Nations (UN) international conference in 2012 in an attempt to guide the path to sustainable development in the world after 2015 [8]. With 17 SDGs and 169 targets, this agenda replaces the narrower Millennium Development Goals (MDGs) and incorporates two main features: “Universality (applies to all countries and populations)” and “commitment to leaving no one behind” [9]. These goals and targets pose implementation challenges for both underdeveloped and developed countries; however, these challenges differ regarding their magnitude in different countries and the ambitions of each country. Moreover, the magnitude of these challenges vary depending on the country’s present state, its differing responsibilities, available resources, and capacity to meet these goals [8]. Regarding SRHR, between 10 and 69% of women globally report intimate partner violence or other physical violence at least once in their lifetime and about 0.3% to 12% of them report sexual violence by non-partners. This figure is even higher in LMICs where the prevalence goes up to 75% for intimate partner violence in some countries [10]. Consequently, due to the persistence of social problems including poverty, health, education and gender issues, LMICs are not on track to meet many of the SDGs [11]. The SDGs require substantial resources across several sectors including “achieving healthy lives and wellbeing for all ages” (SDG 3) and “achieving gender equality and empowering all women and girls” (SDG 5) [12]. There remains significant work to be done to adequately address the complex sociocultural issues that serve as persistent barriers to achieving gender equality and female empowerment, particularly as it pertains to the sexual and reproductive health of girls and women in LMICs.

### Integration of services related to sexual reproductive health and rights (SRHR) and HIV

Integrating the services for SRHR and HIV has been highly promoted globally. This is because it is believed that this strategy not only improves the quality and efficient use of limited resources for policy makers [13], but also promotes the uptake of preventative measures at health facilities. This includes contraceptive and condom use, HIV testing and preventing mother-to-child transmission through antiretroviral prophylaxis. At an individual level, integration of services offers client-centered care which benefits both healthcare provider and client alike [14, 15]. This constitutes an important approach which benefits the macro, meso and micro levels, from the policy makers at the top, to the intermediate health systems, and to the individuals at the service delivery level [16]. In practice, these integrated services (some of which include laws and policies to reduce stigma related to abortion and pregnancy, contraceptive use and counseling) help to prevent both unwanted pregnancies and sexually transmitted infections including HIV [17]. Offering integrated services is particularly important in countries with high prevalence of unwanted pregnancies and HIV, which is the case in most African countries [13]. A significant number of young women who are HIV positive have been shown to continue to engage in risky sexual encounters [18], largely due to systemic inequities and disparities in the social determinants of health including lack of education and poverty [19]. This emphasizes the need to strengthen education, policies, programs, and services targeting young, HIV-positive women in these countries. In order to prioritize SRHR and HIV services to meet SDG 3 and 5, it is vital to strengthen the already low-resourced health systems and ensure essential health equity and quality of care [20]. This will not be possible without first addressing the unique, contextual sociocultural factors that often act as barriers to receiving care. This includes gender discrimination and the societal norms and values concerning individuals’ decision making capacity as it pertains to fertility, sexual orientation or marital status [21]. Similarly to what was observed during the COVID-19 pandemic, HIV increases the strain on the health system and heightens the risk of sexual-based and gender-based violence, especially in LMICs. This increases the urgency of addressing this human right and public health problem in this setting [10, 21].

### Barriers to implementation

Despite the importance of this three-tiered approach, its contribution towards achieving Universal Health Coverage (UHC) and its support by international health policies [16], legal/policy level interventions and national strategies have not been fully addressed in LMICs [22]. The common implementation problems include delayed or incomplete integration of high-level systems function in most sub-Saharan African countries including separate financing streams for SRHR and HIV programs and services, and inadequate health worker training, supervision and retention [16]. Moreover, the inequalities related to SRHR in LMICs have increased particularly for adolescent girls and young women. Factors including interrupted education, poor health services, insecure family financing, and social values and norms that do not support women’s reproductive health autonomy results in women facing more SRHR risks [21].

Regarding economic challenges, so far in most LMICs, there have been no financial estimates of additional resources needed to attain the SDGs [23]. This is aligned with the notion of underfinancing of concurrent development costs by many LMICs which results in the neglect and poor progress of many programs and projects [24]. In most of these countries, critical sexual and reproductive health (SRH) services including safe abortion and oncology services for reproductive cancers are known to be poorly available yet continue to be typically excluded in the Essential Package of Healthcare Services. This leads to a persistent burden of out-of-pocket expenditure and pervasive gender-based inequalities in accessing SRH services [25]. Implementing new policies to assist healthcare financing in low-income countries particularly remains very limited. This is mainly because the taxable population is small given that only a minute proportion of the population’s workforce live in urban areas and work in formal taxable employment sectors. This results in these nations having very limited resources to meet this overwhelming demand [26].

### Suggested strategies for implementation

There remains a significant unmet gap to achieving optimal SRHR and HIV interventions in LMICs which requires urgent attention and innovative solutions [27]. To address these gaps, improving access to essential health services and ensuring financial protection are required to ensure that no girl or woman is forgotten. We therefore suggest the implementation of three key interventions. First, individual and collective capacity building among women and girls should be a primary focus. Second, there is a need for strong intersectoral collaboration, both within and between sectors [1]. Third, policy makers must ensure secure accountable leadership, governance, and enhancement of national and international financing [1].

It is evident that sexual and reproductive health education remains lackluster in sub-Saharan Africa. For example, in 2014, only a third of women aged 15–24 in the region accurately understood how HIV is spread [2], indicating a critical inadequacy pertaining to sexual and reproductive health education targeted to youths. This may be attributed to pervasive stigma and discrimination related to the topic of positive HIV status in sub-Saharan Africa, particularly towards those who are younger, are not educated and who are of lower socioeconomic rank [28]. At the individual level, capacity building can be achieved by improving the accessibility of information and educational programs for women and young girls regarding SRHR and HIV [2].The promotion of self-care is an important strategy to help improve disease outcomes [29]. Providing educational resources would increase awareness about these issues and would allow women to better understand the related health consequences associated with unprotected intercourse and HIV.

Health promotion can include a focus on educating women about family planning including provision of condoms and birth control, educating about safe intercourse and contraceptive options, and ensuring that reproductive health services are accessible. Integrating these SRH services with HIV programs is important given the high rates of sexual activity and unintended pregnancy in women with HIV in sub-Saharan Africa [4, 5]. Education and support to perform self-screening, testing and self-management for HIV is vital for capacity building. This preventative, upstream approach focused on health promotion would promote sustainable behavior change to empower young girls and women to achieve greater autonomy in their sexual and reproductive health decisions. At the collective level, focus should be on reducing stigma around accessing SRH and HIV services, strengthened through intersectoral collaborations amongst government, community health centers, religious groups, and schools. This would help to integrate federal public health messaging into culturally relevant discussions about SHRH and HIV and is an important strategy to increase capacity building collectively. The use of media advertisements on television and in newspapers is also an effective strategy to rapidly increase health promotion campaigns, shown to be successful in previous HIV education [30, 31] and maternal health work in the region [32–34]. These strategies would help improve women’s engagement and autonomy over their health, thereby reducing gender inequalities [27] and strengthening both individual and collective capacity building through intersectoral collaborations.

At the policy level, improved prioritization and striking a better balance between the ambitions of LMICs and what is realistically attainable is required in order to improve underfinancing [35]. Funding allocations to the SDGs in these countries, including funding from donor countries, must also increase significantly to sustain the decade ahead [23, 35, 36]. Most of these nations are unable to extend assistance to populations who reside in rural settings [26]. Community health financing should be considered as an important option to support the health costs for the various subgroups, including low-income and rural-dwelling residents within LMIC populations. Community health financing strategies, particularly the use of mutual health organizations (MHOs), have become successfully integrated into the health economics approach in several Sub-Sahara African countries, including Ghana, Benin, Rwanda, Senegal and Tanzania [37]. It is important that community health financing strategies are context-dependent and culturally appropriate and reflect federal health funding goals [38]. Other strategies include rural health insurance and community involvement in user-fee management [26]. Intersectoral collaboration is vital for the effective drafting and coordination of actions and for the allocation of required financial resources, and should be at the forefront of all future interventions [1].

## Conclusion

There is still a long way to go for women and girls living with HIV to have safe and satisfactory sexual and reproductive health experiences. This pertains especially to most African countries where resources are limited and gender norms that perpetuate inequalities persist [25]. Integrating SRHR and HIV programs and services is paramount to reduce gender inequalities and can be made possible through intersectoral collaboration of national and international agencies and support through foreign aid for LMICs [4, 23, 35, 36]. Interventions on the SDGs have been proven to be successful, and there is evidence that even the poorest country can reach some level of universality. Due to the limited resources in most African countries, every country will need to initiate their own strategic planning, prioritize actions, allocate realistic funding to priority goals, and identify other local financing streams in order to advance towards universal health coverage and achieve SDG 3 and 5 [23, 35].

## Data Availability

Not applicable.
